# Competing Mortality, Not Breast-Cancer Mortality, Explains the Survival Deficit of Breast Cancer After a Prior Non-Breast Cancer: A SEER Competing-Risks Analysis

**DOI:** 10.3390/medicina62101975

**Published:** 2026-10-13

**Authors:** İsmail Zihni, Burcu Zihni, İmren Mutlu, Demet Sarıdemir Ünal, Mehmet Zafer Sabuncuoğlu

**Affiliations:** 1 Department of General Surgery, Faculty of Medicine, Akdeniz University, 07070 Antalya, Türkiye; dsaridemir@akdeniz.edu.tr (D.S.Ü.); mehmetzafer@akdeniz.edu.tr (M.Z.S.); 2 Department of Cardiology, Antalya City Hospital, 07080 Antalya, Türkiye; burcuzihni@yahoo.com; 3 Department of Radiology, Faculty of Medicine, Akdeniz University, 07070 Antalya, Türkiye; imrenmutlu@akdeniz.edu.tr

**Keywords:** second primary breast cancer, competing risks, cause of death, cardiovascular mortality, SEER

## Abstract

*Background and Objectives:* Women who develop breast cancer after a previous cancer of another site have poorer overall survival, yet it is unsettled whether the breast cancer itself is more lethal or whether the deficit reflects competing causes of death in an older, previously treated population. We decomposed this survival deficit by cause of death, defining every endpoint identically across groups. *Materials and Methods:* Using the Surveillance, Epidemiology, and End Results (SEER) database (17 registries, 2010 to 2020), we studied women with invasive breast cancer as a single primary (*n* = 473,073), as a second or later primary after a non-breast index cancer (second primary breast cancer, SPBC; *n* = 26,832), and as a second breast primary (*n* = 40,252). Cause of death was assigned from the SEER cause-of-death-to-site recode, applied identically across cohorts. Cohorts were compared using cause-specific and Fine–Gray models weighted by an inverse-probability propensity score for age, race and ethnicity, subtype, grade, stage, nodal status, year, marital status, income, and rurality. *Results:* Breast-cancer mortality was not higher in SPBC than in single-primary disease, and was if anything lower (10-year cumulative incidence 12.0 versus 13.6 percent; weighted cause-specific hazard ratio 1.06). The deficit fell almost entirely to competing, non-breast mortality (35.3 versus 13.5 percent). In a five-way decomposition, cardiovascular disease was not the leading competing cause; non-cancer causes and the index cancer each accounted for more deaths. Once patient factors were balanced, cardiovascular and other non-cancer hazards were near unity (1.05 and 1.07), leaving further cancer death as the main excess. *Conclusions:* A breast cancer arising after a non-breast cancer is not intrinsically more lethal. Its survival deficit is competing mortality, driven mainly by further cancer death and, to a smaller and largely age-explained degree, by cardiovascular and other non-cancer causes.

## 1. Introduction

As cancer survival improves, a growing share of patients live long enough to be diagnosed with a second, unrelated cancer, and breast cancer is among the most common second primaries in women who have already survived another malignancy [1]. A natural question is whether a breast cancer that follows an earlier cancer behaves worse than one arising de novo. Across registry studies, a consistent pattern has emerged: overall survival is poorer, whereas breast-cancer-specific survival lies much closer to that of women with a single breast cancer. In a large SEER analysis, Wang and colleagues reported modestly higher breast-cancer-specific mortality after a prior malignancy (hazard ratio 1.11), an excess they found to be concentrated in women whose first cancer had been treated with both chemotherapy and radiotherapy [1].

The mirror-image situation, breast-cancer survivors who later develop a cancer of another site, is a different exposure with its own prognosis and has been studied separately [2]. Single-site work on breast cancer after a specific first cancer has nonetheless reached the same headline: for breast cancer following gastric cancer, overall survival was worse, cancer-specific survival was not, and a Fine–Gray analysis of breast-cancer death returned a subdistribution hazard ratio near unity [3].

This recurring divergence between overall and cancer-specific survival is itself informative. If these women are not dying of breast cancer at a higher rate, their excess deaths must fall to competing, non-breast causes, and the useful question becomes which causes, and whether any of them belong to the second cancer rather than to the population that develops it. Cardiovascular disease is an obvious candidate, both because it is a leading non-cancer cause of death among cancer survivors [4,5,6,7,8,9] and because prior anthracycline chemotherapy and thoracic radiotherapy are recognized cardiotoxic exposures [10,11,12,13]. Studies of second primary breast cancer have generally stopped at multivariable adjustment of the cancer-specific endpoint [14], without partitioning the competing mortality by cause.

Decomposing the cause of death in this setting is not simply a matter of counting. In SEER, the cause-specific death classification is applied differently to single and to multiple primaries, so a death attributed to breast cancer in a woman with one tumor may be attributed elsewhere in a woman with several, a discrepancy capable of distorting the very comparison of interest [15,16]. We therefore built every endpoint from the cause-of-death-to-site recode, applied identically in all groups, and used a competing-risks framework to ask what women with a breast cancer after a non-breast cancer actually die of, and whether their excess mortality, cardiovascular death in particular, is intrinsic to the breast cancer or a reflection of the older, previously treated population in which it arises.

## 2. Materials and Methods

### 2.1. Data Source and Study Population

We used the SEER Research Data, 17 Registries, November 2025 Submission (National Cancer Institute; https://seer.cancer.gov, accessed on 10 July 2026), which covers approximately 27 percent of the United States population. Women with a microscopically confirmed invasive breast cancer diagnosed between 2010 and 2020 and with a known age at diagnosis were identified and classified by the SEER multiple-primary sequence number into three groups. Women whose breast cancer was their only recorded malignancy formed the single-primary comparator (BCa-1). Using a person-level linkage to each woman’s earliest recorded primary, women whose breast cancer was a second or later primary were separated into those whose first cancer was at a non-breast site, which formed the exposed group (second primary breast cancer, SPBC), and those whose first cancer was itself a breast cancer, which we retained as an internal control (second breast primary). Women whose index cancer was diagnosed before the registry coverage window, and who therefore could not be linked, were excluded from the primary comparison and were quantified in the Results.

By construction, the single-primary comparator is conditioned on never developing a later primary during follow-up. This is a recognized source of survivor selection, and we return to its likely direction and magnitude in the Discussion.

### 2.2. Covariates

For each breast tumor, we extracted age, race and ethnicity, molecular subtype derived from estrogen-receptor, progesterone-receptor, and HER2 status, histologic grade, anatomic stage, regional nodal status, year of diagnosis, and receipt of surgery, radiotherapy, and chemotherapy. Because the diagnosis window spans three SEER coding systems, grade was harmonized across the 2018 site-specific and pre-2018 systems, and stage and nodal status were harmonized across the successive American Joint Committee on Cancer and summary-stage schemes. As proxies for comorbidity and access to care, we additionally extracted marital status, county-level median household income, and the rural-urban continuum. For the index cancer, we recorded its site, its summary stage, its first-course radiotherapy and chemotherapy, and the interval in years between the index and the breast diagnosis.

### 2.3. Outcomes

The endpoint of interest was death by cause. To avoid an artifact specific to this design, we did not use the SEER cause-specific death classification, which assigns cause differently for single and for multiple primaries, but instead built every cause of death from the SEER cause-of-death-to-site recode, which records the anatomic site or disease category of the underlying cause identically regardless of sequence number [15,16]. Each death was assigned to one of five mutually exclusive causes: breast cancer; the index cancer, defined as a cancer death whose site matched the woman’s own index-cancer site; other cancer; cardiovascular disease, comprising diseases of the heart and cerebrovascular disease, with a broader definition adding hypertensive, aortic, and atherosclerotic causes in sensitivity analyses; and other non-cancer causes. The complete mapping of cause-of-death-to-site values to these five categories is provided in Supplementary Table S1 and was applied identically to all three cohorts.

### 2.4. Statistical Analysis

Cause-specific cumulative incidence was estimated with the Aalen-Johansen estimator [17]. To compare cohorts while balancing measured differences, we fitted a logistic propensity score for SPBC versus single-primary membership on the covariates listed above, including the sociodemographic proxies, and formed weights for the average treatment effect in the treated [18]. Associations with each cause of death were then estimated on two complementary scales: a cause-specific Cox model, which is the appropriate quantity for an etiologic question, and a Fine–Gray subdistribution model [19,20], which describes absolute risk in the presence of competing events. Both were weighted and used cluster-robust variance. For tractability, the Fine–Gray models were fitted on a random four-to-one control-to-case subsample of approximately 107,000 controls; estimates were stable across random seeds. Rather than a formal test of proportional hazards, which is uninformative at this sample size, we report cause-specific hazard ratios within three follow-up windows, 0 to 2, 2 to 5, and 5 to 10 years. Sensitivity analyses used overlap weights [21] and the broader cardiovascular definition, and we computed E-values [22], interpreting each as a bound on the strength an unmeasured confounder would need rather than as evidence that such a confounder exists. As exploratory analyses, we described the composition of mortality by the type and stage of the index cancer and by its first-course treatment and the interval to the breast diagnosis. These analyses were planned a priori but not formally registered. Reporting follows the STROBE guideline for observational studies, and a completed checklist is provided as Supplementary Materials. Analyses used Analyses were performed in R version 4.5.1 (R Foundation for Statistical Computing, Vienna, Austria), using the survival package (version 3.8-3) for the Fine-Gray subdistribution models, and in Python version 3.13, using lifelines (version 0.30.3) for Aalen-Johansen estimation and cause-specific Cox models and scikit-learn (version 1.9.1) for propensity-score estimation.

## 3. Results

### 3.1. Cohort

**Figure 1:**
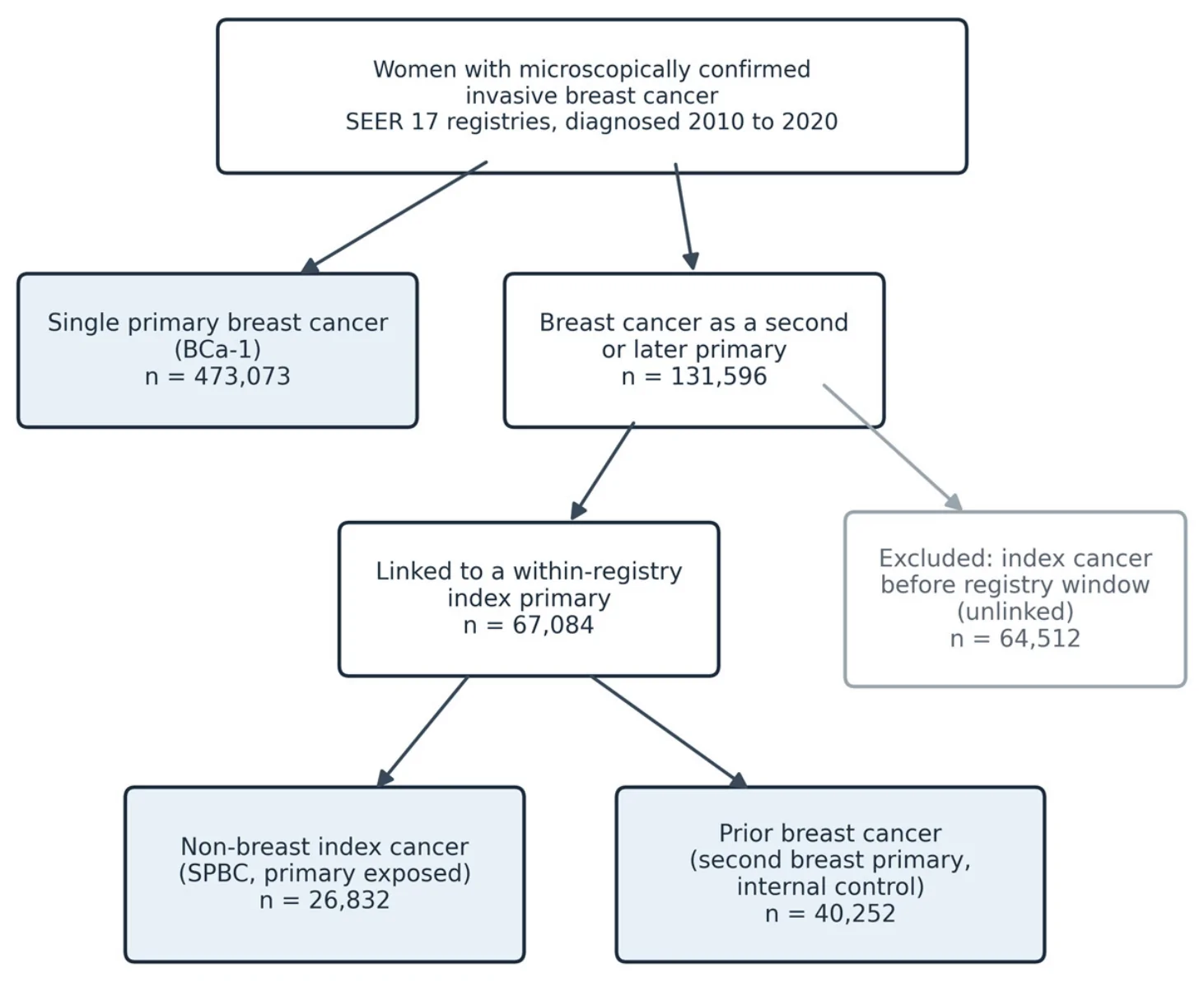
Study flow diagram. Derivation of the three analytic cohorts from women with microscopically confirmed invasive breast cancer in SEER, showing the single-primary comparator (BCa-1), the linkage of second-or-later primaries to a within-registry index cancer, the split into the SPBC-exposed group and the second-breast-primary internal control, and the excluded unlinked cases.

Among women with invasive breast cancer diagnosed between 2010 and 2020, 473,073 had a single primary, 26,832 had a breast cancer that followed a non-breast index cancer (SPBC), and 40,252 had a second breast primary. A further group whose index cancer predated the registry coverage window could not be linked and was excluded from the primary comparison (Figure 1). Women in the SPBC group were older, and their tumors were modestly more often node-negative and of favorable subtype, differences that motivated the propensity weighting (Table 1). The median follow-up was 78, 64, and 68 months in the single-primary, SPBC, and second-breast-primary groups. The median interval between the index cancer and the breast diagnosis was 5.0 years (interquartile range, 2 to 9).

### 3.2. What These Women Die Of

**Figure 2:**
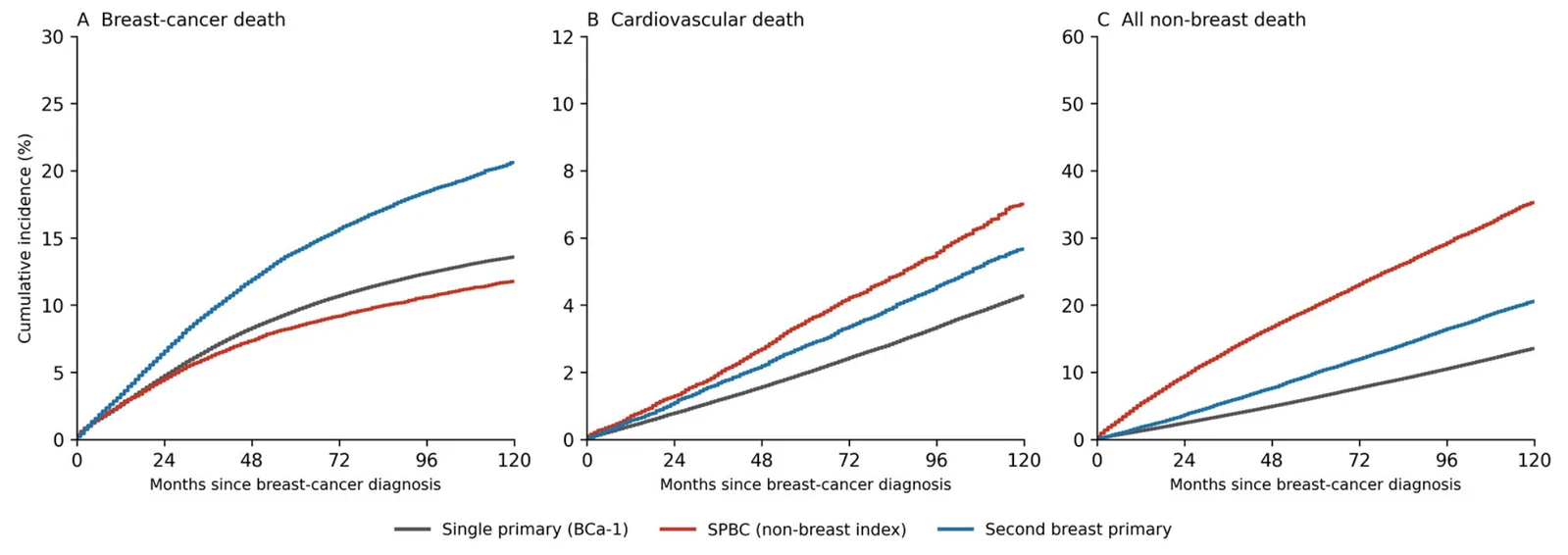
Cause-specific cumulative incidence of death by cohort: (**A**) breast-cancer death, (**B**) cardiovascular death, (**C**) all non-breast-cancer death, in single-primary breast cancer, second primary breast cancer after a non-breast index cancer, and second breast primary. Aalen-Johansen estimator. *Y*-axis scales differ across panels. Numbers at risk at 0, 2, 4, 6, 8, and 10 years were, for single-primary breast cancer, 473,073/432,470/361,733/260,566/176,437/105,564; for SPBC, 26,832/23,070/17,899/11,715/7240/3778; and for the second breast primary, 40,252/35,958/28,261/18,980/11,932/6636.

Ten-year cause-specific cumulative incidence made the central pattern plain. Breast-cancer death was not higher in the SPBC group than in single-primary disease; it was slightly lower (12.0 versus 13.6 percent). The entire survival deficit fell to competing non-breast mortality, which was more than twofold higher (35.3 versus 13.5 percent). The internal control pointed the other way: women whose earlier cancer had also been breast cancer showed clearly higher breast-cancer death (20.8 percent), as expected when two breast cancers contribute to the same endpoint, confirming that the flat breast-cancer mortality in SPBC is specific to the exposure and not a property of any second cancer (Figure 2).

### 3.3. Cardiovascular Disease Is Not the Leading Competing Cause

Partitioning the competing mortality by cause corrected an impression that a two-way split can give rise to. Among the SPBC group, the ten-year cumulative incidence was 12.6 percent for other non-cancer causes, 8.5 percent for the index cancer itself, 7.2 percent for other cancers, and 7.0 percent for cardiovascular disease (Supplementary Table S4). Cardiovascular disease was thus the smallest of the competing causes, not the largest. It appears prominent only when non-cancer deaths are kept as a single block while cancer deaths are separated by site. Because the cohort is defined by a prior cancer, an excess of further cancer death is expected almost by construction; the parts that are not tautological are the non-cancer and cardiovascular components, and it is those we examine on the hazard scale.

### 3.4. Adjusted Hazards

**Figure 3:**
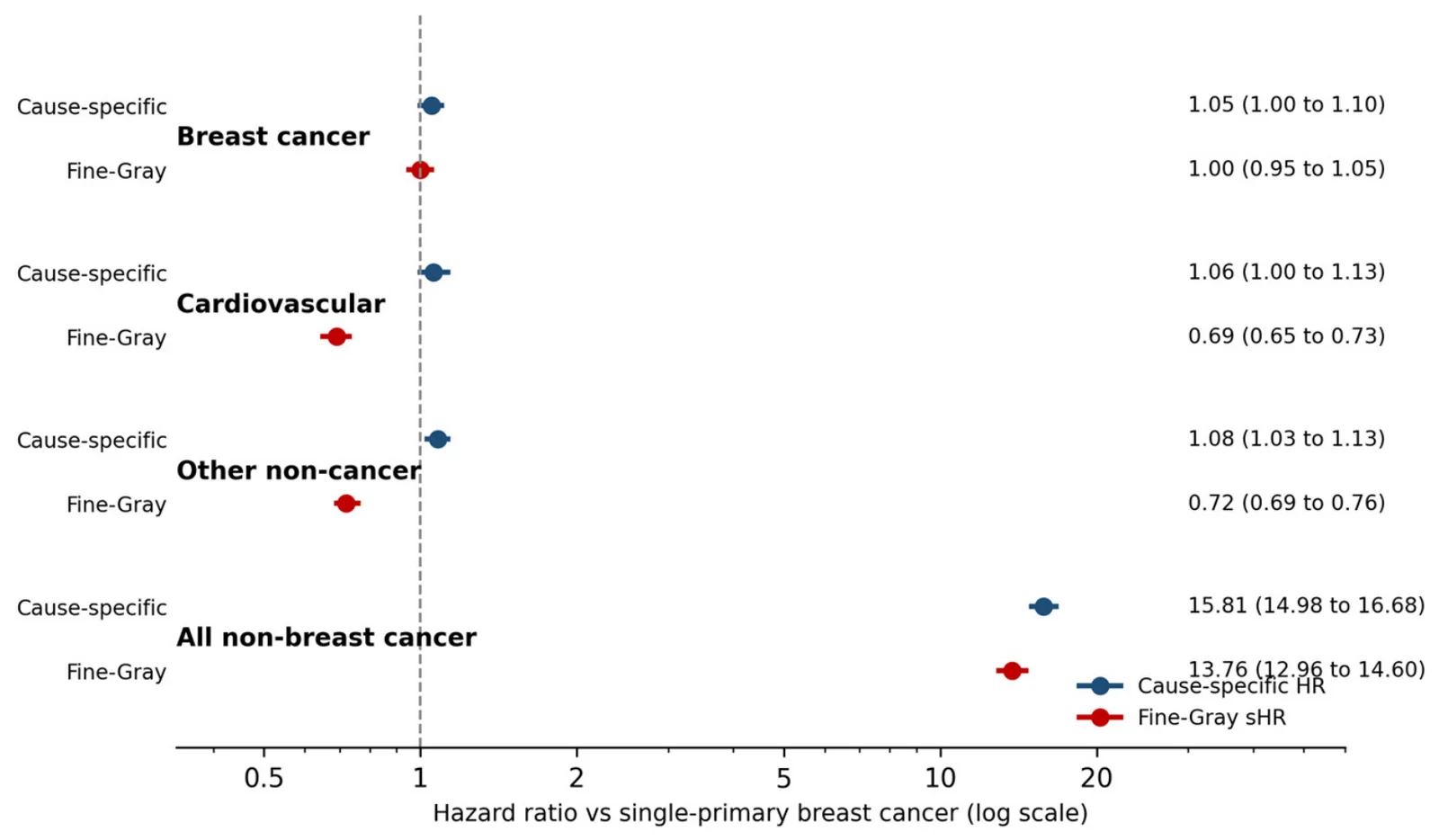
Forest plot of Table 2: cause-specific and Fine–Gray subdistribution hazard ratios for SPBC versus single-primary breast cancer on the log scale, reference line at 1.

Table 2 gives the weighted associations on both the cause-specific scale, which answers the etiologic question, and the Fine–Gray scale, which describes absolute risk (Figure 3). For breast-cancer death, the cause-specific hazard ratio was 1.05 (95% CI 1.00 to 1.10) and the subdistribution hazard ratio was 1.00 (0.95 to 1.05); the absolute risk of dying of breast cancer was therefore no different in the two groups, and any cause-specific excess was at most a few percent. For cardiovascular death, the cause-specific hazard ratio was 1.06 (1.00 to 1.13), its confidence interval reaching unity, so no independent cardiovascular excess was demonstrable once measured patient factors were balanced; the subdistribution hazard ratio was 0.69 (0.65 to 0.73), meaning the absolute cardiovascular burden was in fact lower, because these women more often died of cancer first. Other non-cancer deaths carried a small cause-specific excess (1.08, 1.03 to 1.13). The only large signal was cancer death: taken together, non-breast-cancer death had a cause-specific hazard ratio of 15.81 (14.98 to 16.68), consistent with the expected consequence of having had a prior cancer.

### 3.5. Timing of the Hazards

Because a formal test of proportional hazards is uninformative at this sample size, we examined the cause-specific hazard ratios within follow-up windows. For breast-cancer death, the ratio was flat early and drifted upward only late (1.01 at 0 to 2 years, 1.08 at 2 to 5 years, 1.20 at 5 to 10 years), the opposite of what an intrinsically more aggressive tumor would have produced. For cardiovascular death, no window showed a consistent excess (0.93, 1.18, and 1.03), and in particular there was no early signal such as acute treatment cardiotoxicity would generate.

### 3.6. Type and Stage of the Index Cancer

The competing-mortality excess did not depend on the index cancer being advanced or aggressive. Grouping index cancers by expected behavior, ten-year non-breast mortality rose from 23.0 percent for indolent sites (thyroid, melanoma, corpus uteri) through 39.0 percent for intermediate sites to 47.1 percent for aggressive sites, against 13.5 percent in single-primary disease, yet breast-cancer death stayed at or below the single-primary level in every stratum (Supplementary Table S2). The same held for summary stage, available for 81 percent of the group: even when the index cancer was localized, non-breast mortality was more than double that of single-primary disease (29.5 versus 13.5 percent), while breast-cancer death was not elevated (10.5 percent), rising monotonically to 52.0 percent when the index cancer was distant (Supplementary Table S3). That the excess persists at the lowest index stage indicates it is not simply advanced index disease running its course.

### 3.7. Treatment and Interval of the Index Cancer

We looked directly for the anthracycline and thoracic-radiation mechanism that has been proposed. Within the SPBC group, first-course radiotherapy was recorded for 20 percent of index cancers and chemotherapy for 24 percent. Breast-cancer mortality was identical whether or not the index cancer had received chemotherapy and radiotherapy (10-year cumulative incidence 11.8 percent in both), so we did not reproduce the concentration of breast-cancer risk in doubly treated first cancers reported previously. Cardiovascular mortality was, if anything, lower in women whose index cancer had been treated with both modalities (5.5 versus 7.6 percent), a pattern consistent with confounding by indication rather than treatment cardiotoxicity, and it rose with the interval since the index diagnosis (5.8 percent under five years, 8.0 percent at five years or more), as expected with aging. These analyses use SEER first-course treatment fields, which are incomplete, and are exploratory.

### 3.8. Sensitivity Analyses

The findings were stable. Overlap weighting and the broader cardiovascular definition left the estimates essentially unchanged. Defining breast-cancer death from the cause-of-death-to-site recode rather than the SEER cause-specific classification lowered breast-cancer mortality in all three cohorts, as expected once the sequence-dependent coding was removed, and slightly widened rather than narrowed the competing-mortality gap. For the small residual cardiovascular cause-specific hazard, the E-value was modest, meaning an unmeasured confounder of ordinary strength, such as smoking or hypertension, would suffice to account for it; we read this as a bound on what such a confounder would need to do, not as evidence that one is present.

## 4. Discussion

In this population-based competing-risks analysis, a breast cancer arising after a non-breast cancer was not a more lethal breast cancer. On the absolute scale, the risk of dying of breast cancer matched that of single-primary disease (subdistribution hazard ratio 1.00), and on the etiologic scale, any excess was at most a few percent (cause-specific 1.05). The poorer overall survival of these women was, in its entirety, due to competing, non-breast mortality.

Our breast-cancer estimate sits close to the modestly higher cancer-specific mortality that Wang and colleagues reported in an overlapping SEER population (hazard ratio 1.11) [1,23]. We therefore replicate that figure rather than overturn it; what we add is to set it beside the competing mortality and to show that, in absolute terms, it corresponds to no excess breast-cancer death. For breast cancer after gastric cancer, Sanli and colleagues likewise found worse overall but preserved cancer-specific survival, with a subdistribution hazard near unity [3], and the same divergence recurs across the second-primary literature.

The cardiovascular findings turn on which quantity answers which question. On the cause-specific scale, which is the one relevant to an etiologic effect, the cardiovascular hazard ratio was 1.06 with a confidence interval reaching unity once sociodemographic proxies were added to the propensity model, so no cardiovascular effect independent of measured patient factors was demonstrable. On the subdistribution scale, the absolute cardiovascular burden was in fact lower, because these women more often died of cancer first. The small residual cause-specific signal is most plausibly unmeasured comorbidity, since SEER does not record smoking, blood pressure, or cardiac function [24,25]; the corresponding E-value shows that a confounder of ordinary strength would suffice to explain it, and we read that value as a bound on what such a confounder would need to do, not as evidence that one is present.

We were also able to test the proposed cardiotoxic mechanism directly. If prior anthracyclines or thoracic radiation were driving the pattern, breast-cancer and cardiovascular death should concentrate in women whose index cancer had received both chemotherapy and radiotherapy, and cardiovascular death should appear early. Neither was seen. Breast-cancer mortality was flat across index treatment; cardiovascular death was, if anything, lower in doubly treated women, which points to confounding by indication, and it rose with time since the index diagnosis, as aging would predict. The time-stratified hazards showed no early cardiovascular excess. The competing mortality reads better as the accumulated risk of an older population that has already had cancer than as a late effect of its treatment.

Once patient factors were balanced, the only large excess was further cancer death, the index cancer and new cancers together. This is expected almost by definition in a cohort selected for a prior malignancy, and it persisted even when the index cancer was indolent or localized, so it is not confined to advanced index disease. The genuinely residual component is therefore modest: a small, largely age-explained excess of non-cardiac non-cancer death, and no independent cardiovascular effect.

For survivorship care, the implication is not a new cardiac screening mandate specific to this group. Because the competing mortality is broad and dominated by further cancer and by age-related non-cancer disease, the sensible priorities are continued cancer surveillance and the same guideline-based cardiovascular risk management that suits any older cancer survivor, rather than intensified cardiac testing justified by the second breast cancer as such. Where a woman’s index cancer involved anthracyclines or chest irradiation, established cardio-oncology surveillance already applies [26].

The principal strength of this study is its uniform endpoint. Building every cause of death from the cause-of-death-to-site recode removes the sequence-dependent coding of the SEER cause-specific classification, a recognized hazard when single and multiple primaries are compared [15,16], and the full mapping is reported. A built-in internal control, the use of both hazard scales, and a propensity model extended with sociodemographic proxies further support this reading. The SEER database, which underlies a large body of survivorship research, provides the population coverage such a decomposition requires. Several limitations remain. The single-primary comparator is conditioned on not developing a later primary, which enriches it modestly for early death; because that would tend to raise its breast-cancer mortality, it can only reinforce, not manufacture, the finding that the second breast cancer is no more lethal. SEER captures no comorbidity and its first-course treatment fields are incomplete, so the treatment analysis is indicative only. Death-certificate cause of death is imperfect, and a cardiovascular death and an unrecognized cancer death can be interchanged; the site recode reduces but does not eliminate this, and any residual misclassification would blur rather than create the contrast we report. Finally, women whose index cancer predated the registry window were excluded and may differ in unmeasured ways.

## 5. Conclusions

A breast cancer that arises after a non-breast cancer is not intrinsically more lethal; its absolute breast-cancer mortality matches that of single-primary disease. The poorer overall survival of these women is due to competing, non-breast mortality, driven chiefly by further cancer death and, to a smaller and largely age-explained degree, by non-cancer causes, with no cardiovascular excess independent of patient factors. Their care is best organized around continued cancer surveillance and standard cardiovascular risk management for an older survivor population, rather than around the second breast cancer itself.

## Figures and Tables

**Table 1:** Baseline characteristics of the three cohorts.

Characteristic, *n* (%)	Single Primary (BCa-1)	SPBC, Non-Breast Index	Second Breast Primary
**No. of patients**	473,073	26,832	40,252
**Age, y**			
<50	108,425 (22.9)	1780 (6.6)	4954 (12.3)
50–64	180,802 (38.2)	7570 (28.2)	13,811 (34.3)
65–74	110,322 (23.3)	9041 (33.7)	11,541 (28.7)
75 or older	73,524 (15.5)	8441 (31.5)	9946 (24.7)
**Race/ethnicity**			
White	364,551 (77.1)	22,471 (83.7)	32,026 (79.6)
Black	52,415 (11.1)	2343 (8.7)	4478 (11.1)
Other	51,745 (10.9)	1988 (7.4)	3651 (9.1)
**Molecular subtype**			
HR+/HER2−	321,982 (68.1)	19,469 (72.6)	27,700 (68.8)
HR+/HER2+	49,861 (10.5)	2202 (8.2)	2857 (7.1)
HR−/HER2+	21,003 (4.4)	854 (3.2)	1229 (3.1)
HR−/HER2− (triple negative)	49,885 (10.5)	2448 (9.1)	4149 (10.3)
Unknown	30,342 (6.4)	1859 (6.9)	4317 (10.7)
**Grade**			
I	97,713 (20.7)	6345 (23.6)	9817 (24.4)
II	194,753 (41.2)	11,876 (44.3)	16,832 (41.8)
III to IV	143,124 (30.3)	6457 (24.1)	9360 (23.3)
Unknown	37,483 (7.9)	2154 (8.0)	4243 (10.5)
**Stage**			
I	247,350 (52.3)	15,856 (59.1)	24,122 (59.9)
II	126,945 (26.8)	6260 (23.3)	7240 (18.0)
III	45,873 (9.7)	1849 (6.9)	2438 (6.1)
IV	27,228 (5.8)	1214 (4.5)	2560 (6.4)
Unknown	25,677 (5.4)	1653 (6.2)	3892 (9.7)
**Nodal status**			
Node-negative	249,415 (52.7)	15,599 (58.1)	24,184 (60.1)
Node-positive	122,581 (25.9)	5298 (19.7)	6490 (16.1)
Unknown	101,077 (21.4)	5935 (22.1)	9578 (23.8)
**Surgery**			
No surgery	44,926 (9.5)	3458 (12.9)	5519 (13.7)
Breast-conserving	254,892 (53.9)	15,302 (57.0)	13,342 (33.1)
Mastectomy	171,482 (36.2)	7954 (29.6)	21,136 (52.5)
**Treatment**			
Radiotherapy received	261,181 (55.2)	12,201 (45.5)	11,744 (29.2)
Chemotherapy received	195,892 (41.4)	7262 (27.1)	13,419 (33.3)
**Index cancer (SPBC group)**			
Radiotherapy received	n/a	20.1	n/a
Chemotherapy received	n/a	24.3	n/a
Chemotherapy and radiotherapy	n/a	8.9	n/a
Interval to breast, median (IQR), y	n/a	5.0 (2 to 9)	n/a
**Median follow-up, mo**	78	64	68

Column percentages within each cohort. Surgery percentages omit a small unknown-surgery group (0.4 to 0.6%). Stage was recorded for about 94 percent of patients; the larger nodal unknown reflects the 2016 to 2017 interval, when the SEER combined nodal recode was not populated. Both were retained as explicit categories and balanced by weighting rather than imputed. HR, hormone receptor; HER2, human epidermal growth factor receptor 2. n/a, not applicable.

**Table 2:** Adjusted association of SPBC with mortality by cause compared with single-primary breast cancer (ATT-weighted).

Cause of Death	Crude HR	Cause-Specific HR (95% CI)	Fine–Gray Subdistribution HR (95% CI)
Breast cancer	1.02	1.05 (1.00–1.10)	1.00 (0.95–1.05)
Cardiovascular	1.87	1.06 (1.00–1.13)	0.69 (0.65–0.73)
Other non-cancer	1.76	1.08 (1.03–1.13)	0.72 (0.69–0.76)
Other cancer	8.81	7.08 (6.61–7.59)	5.80 (5.39–6.24)
All non-breast cancer	15.85	15.81 (14.98–16.68)	13.76 (12.96–14.60)

The reference is single-primary breast cancer. Every cause of death was defined from the SEER cause-of-death-to-site recode, applied identically in all cohorts. Both hazard scales use the same inverse-probability weights for the average treatment effect in the treated (propensity score: age, race and ethnicity, subtype, grade, stage, nodal status, year of diagnosis, marital status, county-level income, and rurality). Cause-specific: weighted Cox with competing events censored, cluster-robust variance, full cohort. Fine–Gray: weighted subdistribution model on a random four-to-one control-to-case subsample, cluster-robust variance, stable across random seeds. Index-cancer death is intrinsic to the exposed group and has no single-primary comparator; it is shown by cumulative incidence in Supplementary Table S4 and is combined with other cancers in the last row.

## Data Availability

This is a database study using the SEER Research Data, available to registered users from the National Cancer Institute at https://seer.cancer.gov(accessed on 10 July 2026) and is not redistributable by the authors under the data-use agreement. The SEER*Stat session specifications and the analysis code that produced the tables and figures are available from the corresponding author on reasonable request.

## Supplementary Materials

The following supporting information can be downloaded at https://www.mdpi.com/article/10.3390/medicina62101975/s1: Table S1: Mapping of the SEER cause-of-death-to-site recode to the five analytic causes; Table S2: Ten-year cumulative incidence by index-cancer type; Table S3: Ten-year cumulative incidence by index-cancer summary stage; Table S4: Ten-year cumulative incidence of the five competing causes in the SPBC group; and STROBE checklist.
