# Supplementary material for: Competing Mortality, Not Breast-Cancer Mortality, Explains the Survival Deficit of Breast Cancer After a Prior Non-Breast Cancer: A SEER Competing-Risks Analysis
Source: Medicina (Kaunas). 2026 Oct 13;62(10):1975. doi: 10.3390/medicina62101975 (PMC13706995; doi:10.3390/medicina62101975)
Supplement: Supplementary file 1 [file medicina-62-01975-s001.zip › medicina-4564551-supplementary.pdf]

Supplementary Materials

Competing Mortality, Not Breast-Cancer Mortality, Explains the Survival Deficit of Breast Cancer After a Prior Non-Breast Cancer: A SEER Competing-Risks Analysis

İsmail Zihni, Burcu Zihni, İmren Mutlu, Demet Sarıdemir Ünal and Mehmet Zafer Sabuncuoğlu

Supplementary Table S1. Mapping of the SEER cause-of-death-to-site recode to the five analytic causes

Analytic cause	SEER cause-of-death-to-site values
Breast cancer	Breast
Cardiovascular	Diseases of Heart; Cerebrovascular Diseases (broad definition adds Hypertension without Heart Disease, Aortic Aneurysm and Dissection, Atherosclerosis, Other Diseases of Arteries)
Index cancer	A cancer-site death whose site matched the woman's own index-cancer site
Other cancer	Any other malignant-site death (not breast, not the index site)
Other non-cancer	All remaining non-cancer causes (for example chronic obstructive pulmonary disease, diabetes, Alzheimer disease, pneumonia, accidents, other cause of death, and deaths of unknown cause)

The recode assigns the anatomic site or disease category of the underlying cause identically for single and multiple primaries. The same mapping was applied to all three cohorts.

Supplementary Table S2. Ten-year cumulative incidence by index-cancer type (uniform endpoint)

Group	n	Breast cancer	Cardiovascular	Non-breast (all competing)
Single primary (reference)	473,073	13.6	4.3	13.5
SPBC, indolent index	9,365	10.9	5.5	23.0
SPBC, intermediate index	9,966	12.3	8.3	39.0
SPBC, aggressive index	6,486	12.2	7.0	47.1

Cumulative incidence (percent) at 10 years, Aalen-Johansen estimator. Indolent: thyroid, melanoma, corpus uteri. Aggressive: lung, non-Hodgkin lymphoma, other haematological cancers, pancreas, esophagus, stomach, ovary, liver. The competing-mortality excess is present even in the indolent group, while breast-cancer death stays at or below the single-primary level.

Supplementary Table S3. Ten-year cumulative incidence by index-cancer summary stage (uniform endpoint)

Index-cancer stage	n	Breast cancer	Cardiovascular	Non-breast (all competing)
Single primary (reference)	473,073	13.6	4.3	13.5
SPBC, localized index	13,190	10.5	7.1	29.5
SPBC, regional	4,621	12.0	7.0	41.9

index				
SPBC, distant index	3,201	13.3	5.6	52.0
SPBC, unknown index stage	4,805	13.8	7.8	33.6

Cumulative incidence (percent) at 10 years, Aalen-Johansen estimator. Index summary stage (Combined Summary Stage) was available for 81 percent of the SPBC group. The excess is present even for localized index cancers.

Supplementary Table S4. Ten-year cumulative incidence of the five competing causes in the SPBC group

Cause	10-year cumulative incidence
Breast cancer	11.8
Other non-cancer	12.6
Index cancer	8.5
Other cancer	7.2
Cardiovascular	7.0

Cumulative incidence (percent) at 10 years, Aalen-Johansen estimator, in the SPBC group. Cardiovascular disease is the smallest of the competing causes; non-cancer causes and the index cancer each account for more deaths.

STROBE Statement. Checklist of items for cohort studies

Manuscript: Competing Mortality, Not Breast-Cancer Mortality, Explains the Survival Deficit of Breast Cancer After a Prior Non-Breast Cancer: A SEER Competing-Risks Analysis

An Explanation and Elaboration article discusses each checklist item (Vandenbroucke JP, et al. PLoS Med 2007). The STROBE checklist is available at https://www.strobe-statement.org.

Item	No	Recommendation	Reported in
Title and abstract	1	(a) Design in title/abstract; (b) informative structured abstract	Title; Abstract
**Introduction**			
Background/rationale	2	Scientific background and rationale	Introduction
Objectives	3	Specific objectives / hypotheses	Introduction, final paragraph
**Methods**			
Study design	4	Key design elements	Section 2.1, 2.4
Setting	5	Setting, locations, relevant dates	Section 2.1 (SEER 17, 2010-2020)
Participants	6	Eligibility, sources, selection; matching not applicable	Section 2.1
Variables	7	Outcomes, exposures, predictors, confounders	Sections 2.2, 2.3
Data sources/measurement	8	Sources and methods of assessment; cause-of-death recode mapping	Section 2.3; Supplementary Table S1
Bias	9	Efforts to address bias (uniform endpoint, propensity weighting, immortal-time note)	Sections 2.3, 2.4, 2.1; Discussion
Study size	10	How study size was arrived at	Section 3.1 (full SEER cohort)
Quantitative variables	11	Handling of quantitative variables and groupings	Sections 2.2, 2.4
Statistical methods	12	All methods, confounding, subgroups, sensitivity analyses	Section 2.4
**Results**			
Participants	13	Numbers at each stage; reasons for exclusion	Section 3.1; Figure 1
Descriptive data	14	Characteristics; missing data; follow-up time	Table 1; Section 3.1
Outcome data	15	Number of outcome events by cause	Sections 3.2-3.3; Table 2; Supplementary Table S4
Main results	16	Adjusted estimates, confidence intervals, category boundaries	Table 2; Sections 3.4-3.6
Other analyses	17	Subgroups, interactions, sensitivity analyses	Sections 3.5-3.8; Supplementary Tables S2, S3
**Discussion**			
Key results	18	Summary with reference to objectives	Discussion, first paragraph
Limitations	19	Limitations, sources of bias, direction and magnitude	Discussion, final paragraph
Interpretation	20	Cautious overall	Discussion; Conclusions

		interpretation	
Generalisability	21	External validity	Discussion
**Other information**			
Funding	22	Funding source and role	Funding statement
